# Creatine Use and Thromboembolism Risk in Athletes: A Case Report

**DOI:** 10.7759/cureus.99242

**Published:** 2025-12-14

**Authors:** Osama S Abdalla, Haseeb Mudassir, Hazel Green, Praveenkumar Katarki

**Affiliations:** 1 Acute Medicine, Shrewsbury and Telford Hospital NHS Trust, Telford, GBR

**Keywords:** athletes, creatine supplements, deep vein thrombosis, pulmonary embolism, thrombotic risk

## Abstract

Creatine monohydrate (Cr) is a widely used supplement in the sports and fitness industry, with its popularity continuing to rise. It is well known for its ability to maintain high-energy phosphate levels during intense physical activity, thereby enhancing performance. Documented benefits of creatine supplementation include enhanced muscular development, neuroprotective effects in certain neurodegenerative conditions, and potential cardiovascular advantages. Nonetheless, a growing body of reports has raised concerns regarding possible adverse vascular effects, particularly an increased risk of thrombosis. This case underscores the potential thrombotic risks associated with creatine use and highlights the need to re-evaluate its safety profile. In addition, we provide an updated review of the literature regarding this potentially serious adverse effect.

## Introduction

Creatine monohydrate (Cr) is one of the most widely used and extensively studied dietary supplements in the sports and fitness industry, commonly adopted by both professional athletes and recreational exercisers [1]. Its primary mechanism of action involves the maintenance of high-energy phosphate stores via the phosphocreatine system, which facilitates rapid regeneration of adenosine triphosphate (ATP) during periods of intense physical activity, thereby enhancing performance, strength, and endurance [1]. Beyond its well-established ergogenic effects, creatine supplementation has been associated with multiple physiological benefits, including improved muscle hypertrophy and strength gains [2], neuroprotective properties in certain neurodegenerative conditions such as Parkinson’s and Huntington’s disease [3], and potential cardioprotective effects through enhanced myocardial energy metabolism and endothelial function.

Despite this generally favorable safety profile, emerging evidence has raised concerns regarding possible adverse vascular outcomes, particularly the risk of thrombotic events [4,5]. Although such occurrences are rare, recent case reports and mechanistic studies have suggested that creatine supplementation may influence factors related to coagulation, blood viscosity, and endothelial function, which could contribute to thrombosis in susceptible individuals. This case highlights a possible association between creatine use and venous thromboembolism, emphasizing the importance of critically re-evaluating its vascular safety profile. Moreover, we provide an updated review of the literature regarding this potentially serious adverse effect, aiming to inform both clinical practice and future research on the safe use of creatine supplementation.

## Case presentation

A physically active man in his 20s presented with a three-day history of cramping and swelling in his right calf. He reported waking with a sensation of tightness, cramping, and swelling in the affected limb. The discomfort improved with ambulation during the day but recurred in the evening. He denied any recent trauma to the leg and reported no associated symptoms such as dyspnoea, chest pain, dizziness, fever, or palpitations.

His recent history included a four-hour flight, and he otherwise reported being in his usual state of health. He is a current smoker, consumes alcohol occasionally, and denies the use of anabolic steroids or illicit substances. Notably, he reported regular use of over-the-counter creatine supplements for bodybuilding purposes.

The patient was alert and fully oriented. He exhibited difficulty bearing weight on the right lower limb while ambulating. Cardiovascular examination revealed normal heart sounds without murmurs or additional sounds. Pulmonary auscultation demonstrated clear breath sounds bilaterally, and the abdomen was soft and non-tender with no organomegaly or masses.

Examination of the right lower limb showed swelling in the calf and ankle. The calf was tender. Circumference measured 48 cm on the right and 43 cm on the left (10 cm below the tibial tuberosity). Peripheral pulses were intact on both sides. A small insect bite was seen near the Achilles area of the right ankle, without erythema, warmth, or cellulitis. Investigations (Table 1) showed elevated D-dimer, with normal CBC and C-reactive protein.

**Table 1:** Initial investigations performed in the same day emergency department (SDEC) CRP: C-reactive protein; Hb: Hemoglobin; PLTs: Platelets

Test	Result	Unit	Normal reference range
D-dimer	1869	ng/mL	>500 ng/mL raised
CRP	9	mg/L	(0-5)
Hb	160	g/L	(130-180)
WBC	3.2	10^9^/L	(4.0-11.0)
PLTs	190	10^9^/L	(150-450)

The patient’s coagulation profile was within normal limits. Renal, liver, and bone biochemical profiles were also normal. The electrocardiogram demonstrated a normal sinus rhythm with a heart rate of 90 beats per minute.

Extended coagulation screening revealed a negative lupus anticoagulant and normal levels of protein C, protein S, and antithrombin. Genetic analysis showed no evidence of a prothrombin gene mutation; however, the patient was heterozygous for the Factor V Leiden mutation.

**Figure 1:**
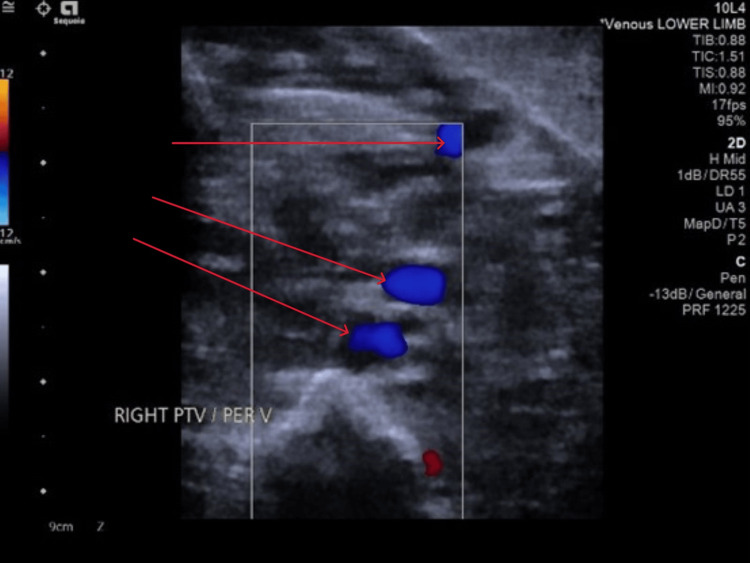
US compression venography of the right lower limb showed evidence of acute thrombus in the peroneal veins of the right calf

Ultrasound Doppler of the right leg demonstrated an acute thrombus within the peroneal veins of the calf (Figure 1). The femoral, popliteal, and posterior tibial veins were patent with no evidence of obstruction. Additionally, normal respiratory variation was observed in the right common femoral vein, indicating preserved proximal venous function.

## Discussion

Cr is one of the most widely utilized and extensively studied dietary supplements in the sports and fitness industry, with its use continuing to rise among both competitive athletes and recreational exercisers. It is well recognized for its role in maintaining high-energy phosphate stores during brief bouts of maximal exertion via the phosphocreatine energy system, supporting rapid adenosine triphosphate (ATP) resynthesis and enhancing exercise performance [1]. The ergogenic benefits of creatine supplementation are well documented, including improvements in muscle hypertrophy, strength gains, and accelerated recovery following high-intensity training [2]. Beyond its musculoskeletal effects, creatine has demonstrated neuroprotective properties in preclinical and clinical models of neurodegenerative diseases, such as Parkinson’s and Huntington’s disease, and has shown potential cardioprotective effects through enhanced myocardial energy metabolism and improved endothelial function [3].

Despite its generally favorable safety profile, emerging literature has raised concerns regarding potential vascular effects, particularly a possible association with thrombotic events [4,5]. This report presents a case of deep vein thrombosis (DVT) temporally associated with creatine supplementation and provides an updated review of the evidence concerning this safety concern.

The mechanisms underlying a potential link between creatine supplementation and thrombosis remain incompletely understood. One proposed mechanism involves the osmotic effect of elevated intracellular creatine concentrations, which promotes water influx into skeletal muscle cells via sodium-dependent transporters [4]. This osmotic shift can alter total body water distribution, potentially resulting in relative dehydration, especially during intense exercise or heat exposure. Such dehydration and hemoconcentration may increase blood viscosity, thereby elevating the risk of thrombotic events [5,6]. Consequently, manufacturers and sports nutrition guidelines consistently emphasize the importance of maintaining adequate hydration during creatine use.

Another proposed mechanism involves creatine’s impact on homocysteine metabolism. Elevated plasma homocysteine is a well-established independent risk factor for vascular endothelial dysfunction and recurrent thromboembolism [7]. Under normal physiological conditions, the body synthesizes creatine primarily in the liver and kidneys. This process accounts for roughly 50-70% of the body’s daily methylation activity and significantly contributes to homocysteine production, as it consumes S-adenosylmethionine during creatine formation [8,9]. Exogenous creatine supplementation suppresses endogenous synthesis via feedback inhibition, which could theoretically reduce plasma homocysteine concentrations [10]. However, evidence regarding the effect of creatine on homocysteine levels remains inconsistent. Some studies have reported that creatine supplementation, particularly when combined with multivitamins, reduces plasma homocysteine concentrations [10]. Conversely, other reports have found that high-dose creatine (>5 g/day) increases plasma homocysteine in individuals with impaired renal function [11]. Furthermore, a controlled study investigating short-term creatine supplementation following acute sprint exercise found that a seven-day regimen did not prevent exercise-induced elevations in plasma homocysteine in healthy adults [12]. This observation is particularly relevant, as it may help explain the potential thrombotic risk associated with transient homocysteine elevations after intense exercise.

Several case reports have described venous thrombotic events in otherwise healthy young men using creatine supplementation, with thrombophilia screening typically yielding normal results [13]. These observations raise important questions regarding potential prothrombotic mechanisms associated with creatine use, despite the absence of conventional risk factors. In alignment with these reports, we present the case of an athletic male who developed DVT while supplementing with creatine. Although the patient had recently completed a four-hour flight, a duration generally considered to confer minimal risk for venous thromboembolism, this factor alone is unlikely to fully account for the event [14]. This case underscores the need for further investigation into whether creatine supplementation may interact with physiological or environmental factors to increase thrombotic risk.

Thrombophilia screening is generally not recommended during the acute phase of venous thrombosis because transient changes, such as reductions in plasma antithrombin, protein C, and protein S, can confound interpretation [15]. Current clinical guidelines advise deferring testing for at least six weeks post-event to allow normalization of acute-phase reactants [16]. Consistent with these recommendations, the patient underwent a comprehensive hematological evaluation after the acute phase, which revealed a negative lupus anticoagulant, normal levels of protein C, protein S, and antithrombin, and heterozygosity for the Factor V Leiden mutation. Although heterozygosity for Factor V Leiden alone rarely results in thrombosis, it serves as a significant predisposing factor, lowering the threshold for clinically relevant events when combined with otherwise minor triggers. This case underscores the importance of considering cumulative risk, where genetic predisposition interacts with situational factors to precipitate events that would otherwise be unlikely in isolation.

Additional case reports have suggested a possible association between creatine supplementation and thrombotic events in young adults. One report described a man in his 20s who developed a pulmonary embolism, with the authors proposing creatine as a potential contributing factor [17]. Another case involved a young male diagnosed with central retinal vein occlusion (CRVO), highlighting the importance of hydration status and the potential vascular effects of creatine supplementation in such pathologies [18].

Conversely, a recent review noted that creatine supplementation might confer vascular benefits, including improved endothelial function and reduced oxidative stress. However, the authors emphasized that these findings are based on a limited number of human studies and called for further clinical research to clarify creatine’s impact on vascular health [18]. Taken together, these observations suggest that, while creatine is generally considered safe, clinicians should remain vigilant for rare but potentially serious thrombotic complications - particularly in young, otherwise healthy individuals engaged in high-intensity exercise or those with additional risk factors.

## Conclusions

Although creatine supplementation is well established for its ergogenic benefits and potential neuroprotective and cardioprotective effects, isolated reports of venous thromboembolism in young, otherwise healthy individuals raise important safety considerations. Any potential thrombotic risk is likely multifactorial, involving osmotic fluid shifts, alterations in homocysteine metabolism, and interactions with physiological or environmental stressors. Clinicians, athletes, and recreational users should be advised on maintaining adequate hydration, adhering to recommended dosing protocols, and monitoring for symptoms of thrombosis - particularly in those with known or suspected prothrombotic risk factors.

Given the limited number of documented cases and inconsistent mechanistic evidence, definitive conclusions regarding causality cannot yet be drawn. Large-scale prospective studies and randomized controlled trials are warranted to elucidate the relationship between creatine supplementation and thrombotic risk, identify susceptible populations, and inform evidence-based guidelines for safe creatine use. In the interim, individualized risk assessment and careful monitoring remain prudent strategies to minimize potential adverse vascular events while maximizing the performance and health benefits of creatine supplementation.
